# Effectiveness of cognitive behavioral group therapy for depression in routine practice

**DOI:** 10.1186/s12888-014-0292-x

**Published:** 2014-10-21

**Authors:** Jens C Thimm, Liss Antonsen

**Affiliations:** Department of Psychology, University of Tromsø, 9037 Tromsø, Norway; Helgeland Hospital Trust, 8607 Mo i Rana, Norway

**Keywords:** Cognitive-Behavioral Group Therapy, Depression, Effectiveness, Routine Care

## Abstract

**Background:**

Previous research has shown that cognitive- behavioral group therapy (group CBT) is an effective treatment for depression. However, the effectiveness of this approach in routine care needs more research. The current study retrospectively examines the outcomes of patients who received group CBT for depression at a psychiatric outpatient clinic between 2003 and 2013.

**Methods:**

Based on patient records, 143 patients were identified as having received the treatment, and 88 patients were included in the outcome analyses. The Beck Depression Inventory (BDI-II) score was the main outcome measure.

**Results:**

The dropout rate was 17.5%. The average BDI-II score decreased from 28.5 to 18.5 from pre-treatment to post-treatment and remained stable at 3-months follow-up. The effect sizes at post-treatment and follow-up were large (d = .97 and d = 1.10, respectively). At post-treatment, 44% of the patients showed a significant improvement in depression, including 30% who recovered; at follow-up, the proportions increased to 57% and 40%, respectively. No predictors of dropout or treatment response were found.

**Conclusions:**

Group CBT for depression can be delivered in routine care settings with good results. However, there are still many patients who drop out or do not benefit from treatment.

**Electronic supplementary material:**

The online version of this article (doi:10.1186/s12888-014-0292-x) contains supplementary material, which is available to authorized users.

## Background

The majority of systematic reviews and meta-analyses have concluded that cognitive behavior therapy (CBT) is an efficacious treatment for depression [1-3]. In various treatment guidelines, e.g., the NICE guideline [4], CBT is therefore recommended as the first-line treatment for depression.

Delivering CBT for depression in a group format is a cost-effective alternative to individual treatment [5,6]. Group therapy may provide further advantages, as patients may benefit from group cohesion and normalization effects and may also be able to use the group as an arena for engaging in behavioral experiments, learning from others and functioning as co-therapists [7,8]. On the other hand, group therapy is not acceptable to some patients, and there is less time allotted and less opportunity to tailor treatment to the individual patient [8].

A meta-analysis of 48 randomized controlled trials (RCTs) by McDermut, Miller, and Brown [9] shows that different forms of group therapy effectively reduce depressive symptoms. The authors found an overall effect size of 1.03 and that CBT was somewhat more efficacious than psychodynamic group therapy. In a review of 34 studies on group therapy for depression, Oei and Dingle [10] also examined measures of cognitions, behaviors and general health in addition to depression severity in their analyses. Based on 13 controlled studies, the authors found an average effect size of 1.11 in favor of group CBT. Analyses of 21 uncontrolled studies showed an average effect size of 1.30 for comparisons between pre-treatment and post-treatment scores. Oei and Dingle [10] concluded that group CBT for depression is as effective as other bona fide treatments as defined by Wampold, Minami, Baskin, and Callen Tierney [11]. With respect to group CBT provided in primary care or in the community, a meta-analysis of 14 randomized controlled trails by Huntley, Araya, and Salisbury [12] showed a significant effect of group CBT over usual care at post-treatment and medium- to long-term follow-up; the standardized mean differences (SMDs) reported by the authors were -.55 and -.47, respectively. The authors further found that individually delivered CBT was superior to group CBT (SMD = .38) immediately after treatment, but not at follow-up. Similarly, the results of the meta-analysis conducted by Cuijpers, van Straten, and Warmerdam [13] suggest that group CBT for depression might be slightly less effective than individual therapy in the short-term. A recent review by Okumura and Ichikura [14] extended previous meta-analyses in several respects by comparing group CBT for depression with different levels of treatment intensity as described in stepped care models for depression [4]. Their meta-analysis of 35 studies showed that group CBT was superior to non-active controls (SMD = −.68) and that there was a small but non-significant advantage of group CBT above middle-intensity interventions (SMD = .21).

Concerns have been raised as to whether the findings from research studies can be generalized to routine clinical practice. In this context, it is common to distinguish between the efficacy and the effectiveness of a treatment [15,16]. Efficacy refers to the results achieved in research trials, whereas effectiveness is understood as the therapy outcome in routine practice. The primary goal of research trials is to establish a causal relationship between a given treatment and an outcome (internal validity). In research trials, participants are often selected patients and are treated by trained therapists who follow treatment manuals strictly, receive regular supervision and whose treatment adherence is closely monitored [15]. In contrast, routine clinical practice is characterized by unselected patients, high therapist caseloads, and flexible use of treatment protocols. It has been suggested that due to strict exclusion criteria, patients participating in clinical trials are not representative of patients typically seen in clinical practice in terms of severity and comorbidity, compromising the generalizability of RCTs (external validity) [17-20]. However, recent studies report only minor differences in clinical characteristics between patients participating in RCTs and patients seen in clinical practice, which may indicate more liberal inclusion criteria in more recent RCTs [21-23]. Due to practical and ethical reasons, randomization of patients to an active or non-active control condition is often not feasible in ordinary clinical settings, and some authors have argued that randomization is not representative of clinical practice [24]. Finally, due to publication bias, the effects of treatment for depression found in research trials may be overestimated [25]. Therefore, research on the effectiveness of treatment in routine clinical practice is needed. Although effectiveness studies do not typically have a control group and are therefore unable to establish causal relationships, they may provide valuable information about a given treatment.

Several studies have investigated the effectiveness of CBT for adult depression in routine practice e.g., [26,27]. Recently, Hans and Hiller [24] conducted a meta-analysis of these studies. To define the clinical representativeness of a study, the authors suggested the following criteria based on the work of Shadish and colleagues [28,29]: (a) non-university setting; (b) referred patients; (c) professional therapists with regular caseloads; (d) flexible structure; (e) no monitoring of treatment implementation; and (f) no therapist training for study purposes. A total of 34 studies (1,880 patients) were included in the analyses. Hans and Hiller [24] found an average pre-post effect size of 1.13 for treatment completers and 1.06 for intent-to-treat analysis in reducing depression severity. There were no significant differences between individual and group therapy in this regard. Effect sizes between 0.67 and 0.88 were found for secondary outcome measures (e.g., dysfunctional cognitions, anxiety). The mean dropout rate was 25% and was significantly higher in individual (on average 42%) than group CBT (17%). Hans and Hiller [24] concluded that outpatient individual and group therapy for depression is effective in routine clinical practice. However, the authors characterized their findings as preliminary as the number of available studies was low and samples sizes were often small.

Thus, the purpose of the present study is to add to the knowledge base about the effectiveness of treatment for depression in routine clinical practice settings. In this study, we retrospectively evaluated the effectiveness of group CBT treatment administered in a specialized psychiatric outpatient clinic; the Beck Depression Inventory (BDI) [30,31] were used to assess patients’ depression severity before treatment, during treatment, after treatment, and at 3-months follow-up. In addition, the current study aimed to investigate the pattern of patient dropout from treatment and differences between patients who benefit from the treatment and those who do not respond to the intervention.

## Method

### Participants

The present study draws from a project that evaluated the effectiveness of a treatment for depression given at the group therapy unit at the Psychiatric Centre of the Helgeland Hospital Trust in Mo i Rana in Norway. The center is a secondary care setting located in a rural area near the polar circle that serves a population of approximately 33,000 individuals. Patients are referred to the clinic primarily by their general practitioner, but other specialized health services can also refer patients to the outpatient clinic.

Using the hospital’s electronic record system, the records of patients who were registered as having received cognitive behavioral group therapy for depression between 2002 and 2013 were reviewed. A total of 143 patients (71% female, mean age =41.6 years, range =20 to 69 years) were identified; the patients had participated in 26 different treatment groups. The dropout rate was 17.5% (25 patients). We defined dropouts broadly as patients who attended the first group session but discontinued the treatment at a later time point. Treatment completers could miss single sessions. For 88 patients (62% of the total sample), 73% female with a mean age of 41.8 years (SD =11.3, range =20 - 68), a pre-treatment and post-treatment or follow-up scores on the BDI were available; these patients were included in the outcome analyses. Further demographic and clinical characteristics of this sample are displayed in Table 1. Until 2006, the patients’ diagnoses were established using the Structured Psychiatric Interview for General Practice SPIFA; [32]. Since 2006, the MINI [33] has been routinely used for diagnoses at the group treatment unit.

**Table 1:** **Demographic and clinical characteristics of the sample**

**Sample characteristics**	**N (%)**
Cohabitating or married	56 (63.6)
Education	
Lower secondary school	16 (18.2)
Upper secondary school	38 (43.2)
Higher education	25 (28.4)
Unknown	9 (10.2)
Working	49 (55.7)
First diagnosis (ICD-10)	
F32 Depressive episode	26 (29.5)
F33 Recurrent depressive disorder	44 (50)
F34.1 Dysthymia	10 (11.4)
Other (bipolar disorder (2), obsessive-compulsive disorder (1), post-traumatic stress disorder (2), adjustment disorder (2), avoidant personality disorder (1))	8 (9.1)
Patients with ≥2 diagnoses	28 (31.8)

### Therapists and treatment

The groups were led by a therapist and co-therapist. The therapists were mainly psychiatric nurses, but other mental health professionals (e.g., psychologists) were also group leaders. During the period studied, all therapists had received formal training in CBT. Prior to treatment and baseline assessment with the BDI-II and the BAI, a member of the group therapy unit met the client for a clinical assessment (if the client had not been diagnosed before), to provide information about the group treatment, to discuss with the patient whether the treatment was suitable for him or her and to determine the patients’ motivation. This clinical assessment period typically lasted approximately 4–5 sessions. Group sessions were closed and comprised 5–7 patients when they started. The treatment initially consisted of 12 weekly sessions, but was later extended to 15 sessions. Each session lasted 120 minutes, including a 15-minute break. The content of the group sessions was based on manuals for the cognitive behavioral treatment of depression that were available in Norwegian [34]. As no single manual was used during the study period, there was some variation in the treatment received by groups. However, the core elements of CBT for depression, such as psychoeducation, behavioral activation, and cognitive restructuring, were central to all treatments. In its current form, the group CBT treatment given at the center is guided by the manual written by Hagen and Gråwe [34], and the elements are psychoeducation about depression (two sessions), self-assertion, interpersonal relationships, and social network (three sessions), resources and pleasurable activities (one session), the cognitive model of depression and cognitive restructuring (eight sessions), and relapse prevention and evaluation of treatment (one session). A patient workbook is used during treatment. Each session has the following structure: 1) review of homework; 2) presentation of topic A; 3) exercise related to topic A - conducted individually, in pairs, or in groups; 4) break; 5) presentation of topic B; 6) exercise related to topic B - conducted individually, in pairs, or in groups; and 7) presentation of homework. (A parts and timing plot detailing the current treatment timeline can be found in the online appendix). Approximately three months after the last treatment session, patients receive a follow-up group session that focuses on treatment evaluation and relapse prevention.

### Measures

The BDI is the main outcome variable in the present study. The BDI [30] and its successor, the BDI-II [31], are widely used, 21-item, self-report inventories designed to assess depression severity. Items are answered on a four-point scale ranging from 0 to 3. The BDI was used at the group therapy unit until the spring of 2009, at which point use of the BDI-II began. Due to the differences between the two versions of the inventory, all BDI scores were converted to BDI-II scores using the adjustment table in the BDI-II manual [31] for comparability. According to the BDI-II manual, the adjustment table is based on a study of psychiatric outpatients and an equipercentile equating method [31]. The Norwegian version of the BDI-II has been shown to have a high internal consistency (Cronbach’s alpha = .91) and an acceptable test-retest reliability (.77) over a three week period [35].

The Beck Anxiety Inventory (BAI) [36] consists of 21 items assessing the severity of anxiety symptoms on a four-point scale ranging from 0 to 3. The Cronbach’s alpha for the Norwegian version of the BAI is .88, and its test-retest reliability over three weeks is .69 [37].

The BDI/BDI-II and BAI were administered to patients at the start of group treatment, at approximately mid-treatment (week 7), at the end of the group treatment, and at 3-months follow-up (Additional file 1).

According to the Norwegian Health Research Act, approval from the Research Ethics Committee is not required for the evaluation of routine service delivery (http://www.regjeringen.no/upload/HOD/HRA/Helseforskning/Helseforskningsloven%20-%20ENGELSK%20endelig%2029%2006%2009.pdf and http://www.regjeringen.no/upload/HOD/HRA/Veileder%20til%20helseforskningsloven.pdf). The Data Protection Official for Research for the Helgeland Hospital Trust was notified of the study.

### Statistical analyses

Differences between subgroups of patients were investigated using χ2 tests for categorical data and ANOVA for quantitative variables. The overall effect of the treatment was examined using multilevel modeling. This approach was considered particularly suited for the current investigation, as the analyses did not require complete data for every subject [38,39]. In the analyses, time was defined as fixed factor, and the BDI-II and BAI scores were the dependent variables. Group membership was defined as a level 2 variable. Random intercepts and slopes were specified. Finally, an autoregressive covariance structure with heterogeneous variances was assumed. Unfortunately, as almost no data were available for patients who dropped out of treatment, intent-to-treat analyses could not be performed. Effect sizes (d) between two time points were calculated by dividing the mean differences in outcome variables by the standard deviations of the differences. Uncontrolled effect sizes were calculated for available data pairs and for a data set in which missing data were imputed. The handling of missing data followed the recommendations of Schlomer, Bauman, and Card [40] and Sterne et al. [41]. To evaluate the pattern of missing data, Little’s [42] MCAR test was used. Missing BDI-II and BAI values were imputed by means of a multiple imputation procedure [43] using the automatic method in SPSS 21.0. The number of imputations was specified as 20, and the range of imputed values was constrained to a minimum of 0 and a maximum of 63. The automatic method uses linear regression as model for scale variables [44]. All available BDI-II and BAI scores were included in the imputation procedure.

To further evaluate treatment success and to categorize patients as recovered, improved, unchanged, or deteriorated, the Jacobson and Truax [45] approach as recommended by Bauer, Lambert, and Nielsen [46] was used. For the BDI, cut-off values for reliable change and recovery have been developed [47]. However, as a the BDI and the BDI-II are not entirely identical instruments, it was decided to calculate cut-off scores for the BDI-II and BAI based on the characteristics of the present sample and existing data from the Norwegian general population [35,37]. Patients who showed no reliable change in their BDI-II scores were classified as “unchanged”. If there was a reliable change in negative direction, the patient was classified as “deteriorated”. Patients showing reliable change in positive direction were classified as “improved”, and if patients’ BDI-II scores were below the cut-off for the normal range in addition, the patients were classified as “recovered”.

## Results

### Analysis of dropouts and representativeness of the outcome sample

As mentioned above, 25 (17.5%) of the 143 patients who started the treatment dropped out. For dropouts, the demographic characteristics of age and sex and the diagnosis were collected and available for analyses. The mean age of patients who dropped out was 38 years (SD =11.4), and 18 (72%) were female. Patients who dropped out attended an average of 4.5 sessions (SD =2.8). Age, sex, diagnosis, and pre-treatment scores of the BDI-II and BAI of treatment completers versus dropouts were compared. There was a tendency (*p* =0 .083) for dropouts to be younger than treatment completers, but no significant differences between completers versus dropouts were found with respect to sex, diagnosis, and BDI-II and BAI scores at pre-treatment. Participants dropped out for a variety of reasons, including the need for inpatient or individual treatment (n =12), symptom reduction (n =3), disagreement with the therapist (n =2), absence due to family problems (n =2), sexual harassment of a group member (n =1), pregnancy problems (n =1), somatic illness (n =1), meeting of an acquaintance in the group (n =1), and unknown (n =2). Fourteen of the dropout patients received an alternative treatment. Treatment completers attended an average of 12.1 session (SD =1.7, range =9 - 15). Approximately one quarter of the participants (27%) attended all sessions.

To examine the representativeness of the sample, treatment completers (n =88) with and without BDI-II scores available at pre-treatment and post-treatment or follow-up were compared with respect to sex, age, diagnosis, and BDI-II and BAI scores at pretreatment. No significant differences were found between treatment completers with and without BDI-II scores, indicating that the patients included in the following analyses are representative of all treatment completers.

### Effect of treatment

The means, standard deviations, and percentage of missing data at the four time points are displayed in Table 2. Little’s MCAR test was non-significant, χ2(72) =66.56, *p* = .66, supporting the assumption that data were missing at random, which is a prerequisite for multilevel modeling and multiple imputation [38,43]. As shown in Table 2, except for the BDI-II at pre-treatment, there were missing data at every time point, ranging from 17% (BDI-II at mid-treatment) to 42% (BAI at post-treatment). Approximately two third of cases (64.3%) had missing data for at least one time point; in total 24.7% of the outcome values were missing. The reasons for missing data could not be determined from the electronic record system.

**Table 2:** **Descriptive statistics for the BDI**-**II and the BAI at pre**-**treatment**, **mid**-**treatment**, **post**-**treatment**, **and follow**-**up**

	**Pre**-**treatment**		**Mid**-**treatment**		**Post**-**treatment**		**Follow**-**up**	
	**M (SD, range)**	**Number of missing data (%)**	**M (SD, range)**	**Number of missing data (%)**	**M (SD, range)**	**Number of missing data (%)**	**M (SD, range)**	**Number of missing data (%)**
BDI-II	28.52 (10.42, 2 – 53)	0 (0)	23.03 (11.24, 0 – 51)	15 (17)	18.53 (11.09, 1–44)	18 (20.5)	18.26 (12.24, 0 – 53)	19 (21.6)
BAI	19.07 (13.09, 0 – 58)	21 (23.9)	17.60 (11.73, 0–54)	33 (37.5)	14.12 (11.43, 0 – 45)	37 (42)	14.74 (12.23, 0 – 46)	31 (35.2)

The average BDI-II scores decreased from 28.5 to 18.5 from pre-treatment to post-treatment and remained stable at follow-up (18.2). Mixed-level analysis showed a significant linear effect of time on depression, F(1, 272,98) =66.26, *p* < .001. The linear effect of time on anxiety was also significant, F(1, 215,58) =8.71, *p* < .01. There were no differences between treatment groups. Effect sizes for the differences between the pre-treatment scores and patients’ scores at the three other time points are shown in Table 3. The table contains effect sizes based on available data in addition to effect size estimations using multiple imputation of missing data. Applying Cohen’s [48] criteria (d = .2: small effect; d = .5: medium effect; d = .8: large effect), the effect sizes for depressive symptoms based on available data at post-treatment (d = .97) and follow-up (d =1.10) indicate a large effect, and the effect sizes for anxiety indicate a moderate effect (d = .52 and d = .50, respectively). There were only minor differences in effect size estimations between those based on actual data versus data including multiple imputations of missing data.

**Table 3:** **Effect sizes (d)**

	**Pre**-**treatment to mid**-**treatment**		**Pre**-**treatment to post**-**treatment**		**Pre**-**treatment to follow**-**up**	
	**Available data** **(N)**	**MI of missing data**	**Available data** **(N)**	**MI of missing data**	**Available data** **(N)**	**MI of missing data**
BDI-II	0.59 (73)	0.53	0.97 (70)	1.00	1.10 (69)	1.07
BAI	0.17 (54)	0.16	0.52 (51)	0.49	0.50 (54)	0.43

*Note. MI* = multiple imputation.

### Treatment response

Application of the Jacobson and Truax [45] formula resulted in cut-off scores indicating a reliable change in symptom severity of 10 for the BDI-II and 10.88 for the BAI. Cut-off scores for the normal range of the BDI-II and BAI were 16.66 and 9.26, respectively. The latter value for the BDI-II is slightly higher than the cut-off scores for the BDI reported by Seggar et al. [47] and others [49], which typically range from 13 to 15. A probable explanation for the difference in the cut-off scores is that the BDI scores are, in general, somewhat higher than the BDI-II scores according to the adjustment table in the BDI-II manual. Patients scoring in the normal range of the BDI-II and BAI at pre-treatment were excluded from the analyses.

Available data on depression severity at post-treatment (n =61) showed that 2 patients (3.3%) had deteriorated, 32 (52.5%) remained unchanged, 9 (14.8%) had improved, and 18 (29.5%) had recovered after treatment. At follow-up (n =63), 1 patient (1.6%) had deteriorated, 26 (41.3%) remained unchanged, 11 (17.5%) had improved and 25 (39.7%) had recovered compared to treatment start. With respect to anxiety (n =39), 1 patient (2.6%) had deteriorated, 26 (66.7%) remained unchanged, 11 (28.2%) had improved, and 1 (2.6%) had recovered at post-treatment. At follow-up (n =43), 3 patients (7%) had deteriorated, 28 (65.1%) remained unchanged, 6 (14%) had improved, and 6 (14%) had recovered.

### Predictors of treatment effects

To investigate the characteristics of treatment responders, patients who showed reliable improvement (including recovery) were compared to the group of patients who had either no significant positive change or had deteriorated at post-treatment. The two groups were compared on all available demographic and clinical characteristics (i.e., age, sex, partner status, education, working, first diagnosis, and number of diagnoses), pre-treatment scores on the BDI-II and BAI, and the number of sessions attended. There was a tendency for patients who benefited from the treatment to have higher scores on the BDI-II at pre-treatment compared to those who did not benefit (32.59 and 28.61, respectively, *p* = .098). However, there were no significant differences on the remaining variables examined between treatment responders and non-responders.

## Discussion

The aim of the present study was to examine the effectiveness of group cognitive behavioral therapy for depression in a routine care setting and to explore predictors of treatment dropout and response. The routine care setting - a rural outpatient clinic - meets Hans and Hiller’s [24] criteria for clinical representativeness.

The results showed a significant reduction in depression and anxiety among patients who received group CBT. The observed treatment gains were maintained at 3-months follow-up. The effect sizes of group CBT for depression were large (d = .97 and d =1.10 at post-treatment and follow-up, respectively) and were similar to the results reported in Hans and Hiller’s [24] meta-analysis of the effectiveness of outpatient CBT for depression (d =1.13), adding further support to their findings. In contrast, the effect of group CBT on the severity of anxiety symptoms was only moderate, suggesting that the treatment effect may be specific to depressive symptoms resulting in more positive outcomes for the targeted problem. In terms of clinical significance, the results showed that approximately 44% of the patients saw a significant improvement in depression severity at post-treatment, including approximately 30% who recovered. At follow-up, the proportion of patients who improved and recovered increased to 57% and 40%, respectively. Thus, a considerable number of patients benefited from the treatment. However, effect sizes in the present study are lower than the results reported from efficacy studies. For example, Teri and Lewinsohn [50] and Neimeyer, Kazantzis, Kassler, Baker, and Fletcher [51] found effect sizes of 1.93 and 1.19 for group CBT for depression, respectively. The lower effect sizes found in this study are in accordance with previous studies showing significantly lower effect sizes for treatment of depression in routine care settings compared to research trials [24,52]. On the other hand, the response rates at follow-up are comparable to those found in efficacy studies or effectiveness studies conducted in university settings. According to Keitner, Ryan and Solomon [53], in efficacy studies, 50-58% of depressed patients respond to and 30-48% recover after psychotherapy. Peeters et al. [54] found a remission rate of 37% after 26 weeks of individual cognitive behavioral therapy for depression using the BDI-II to assess outcomes. Unfortunately, the cut-off values on the BDI-II used to define response and remission vary between studies, making direct comparisons difficult. For example, Peeters et al. [54] used a more conservative BDI-II cut-off score of 10 to distinguish between the normal and clinical range. In the current study, neither demographics nor pre-treatment scores on the BDI-II or BAI predicted treatment response. The finding that age and sex are unrelated to treatment outcomes has been reported previously [55], but some studies have found that older age is associated with a poorer outcomes [55]. There was a tendency (*p* < .10) for patients with higher BDI-II scores at pre-treatment to have greater treatment gains. This finding is in line with the findings of Schindler, Hiller and Witthöft [56]. However, Organista, Munoz and Gonzalez [57], Merrill et al. [26], and Teri and Lewinsohn [50] reported that lower initial BDI scores predicted greater improvement. Surprisingly, treatment response was not predicted by the length of treatment, suggesting that a time frame of 12 sessions may be sufficient.

The dropout rate (17.5%) for patients in the present study was somewhat lower than the rates found in both the Hans and Hiller [49] meta-analysis (24.6%) and the Neimeyer et al. [51] and Peeters et al. [54] studies (23.9% and 28%, respectively); however it was higher than in other investigations, e.g. the Teri and Lewinsohn [50] study (8%). Age, sex, diagnosis, and BDI-II or BAI pre-treatment scores did not predict patient dropout. These results are consistent with previous findings [58,59]. As in the Arnow et al. study [59], there was a statistically non-significant tendency for dropouts to be younger in age.

Unfortunately, in the current investigation, there were only a few variables available to examine as predictors of dropout and treatment response. Other factors that have previously shown predictive value for outcomes in the treatment for depression (e.g., chronicity of problems [55], normal personality traits [60], personality disorders [61], intelligence [55], or attachment style [62]) should, if possible, be included in future effectiveness studies.

As encouraging as the results demonstrating the effectiveness of group CBT for depression - delivered in a specialized routine care setting, mainly by psychiatric nurses - are, too many patients drop out of treatment or do not benefit from treatment. There is a need to improve the treatment of these groups of patients. Because many clinicians overestimate the impact of their interventions [63], monitoring treatment outcomes and providing feedback to therapists may increase the effectiveness of treatment [64]. Systematic assessment of patients’ suitability for this type of treatment may also contribute to higher response rates [65]. Finally, a combination of traditional CBT techniques and newer approaches to CBT (e.g., mindfulness-based CBT [66] or meta-cognitive therapy [67]) may enhance treatment effects.

The strengths of the present study are that a follow-up was included, diagnoses were established using a structured diagnostic interview, and appropriate statistical methods were used. On the other hand, effectiveness research faces challenges and involves limitations that also apply to the present study [27]. Because there was no control group, the observed effects cannot be attributed to the treatment with certainty and may instead be attributed to the passage of time or regression to the mean. No data were collected after patients dropped out of treatment; therefore, intent-to-treat analyses could not be performed. The retrospective design of the current study poses additional problems and may be subject to potential biases. Only information already contained in patient records could be used. There was a high number of missing data points, and the quality of the patient records varied greatly. The exact reasons why data were lost are unknown. The missing data could have been due to clinicians not delivering the instruments to the patients or their failure to record patient results in their electronic record; alternatively, the patients may not have returned the inventories. There is a possibility that therapists may have chosen to not give the inventories to non-responders, which would bias the results. However, the results of Little’s MCAR test suggest that the data were missing at random. Some demographic characteristics (e.g., marital status) were difficult to collect. More importantly, the information on the patients’ use of medication, which was usually prescribed by the patient’s general practitioner, was often inadequate, especially in the first years of the study period. It was therefore impossible to control for use of medication in the analyses, and the possibility that the observed changes in patient outcomes are due to the start of or a change in medication cannot be ruled out. However, in our experience, medication is rarely started or changed during group treatment. As is common in clinical practice, patients were selected for group treatment. Unfortunately, there were no available data for patients who were not offered group treatment or who dropped out before the start of group treatment. Thus, any possible selection bias could not be estimated. To overcome the problems inherent to a retrospective approach, we recommend a prospective design for future studies examining the effectiveness of psychotherapy in ordinary clinical settings. Such future studies could, for example, be conducted in conjunction with routine outcome monitoring [68]. Further, a shortcoming of the present study is that two different versions of the BDI were used. In addition, a follow-up period of three months is too short to make conclusions about the long-term effect of the treatment. Finally, in this study, only symptom reduction was measured; however, gains in well-being and life functioning should also be part of treatment evaluation [69] in future studies.

## Conclusion

In conclusion, the present study demonstrates that group CBT for depression, delivered in routine care settings, has good results in terms of both improvement at the group level and clinical significance at the individual level. However, there are still many patients who drop out of treatment or who do not benefit from treatment.

## Additional file

Additional file 1:
**Parts and timing plot.**
