# Supplementary material for: Effectiveness of cognitive behavioral group therapy for depression in routine practice
Source: BMC Psychiatry. 2014 Oct 21;14:292. doi: 10.1186/s12888-014-0292-x (PMC4209079; doi:10.1186/s12888-014-0292-x)
Supplement: Additional file 1: — Parts and timing plot. [file 12888_2014_292_MOESM1_ESM.docx]

Online appendix

*Parts and timing plot*

| Time line | | Intervention |
| --- | --- | --- |
| Assessment | | b  a |
| Week 1 | | d  1  c |
| Pre-treatment measurement | | 2 |
| Week 2 | | d |
| Week 3 to 5 | | e |
| Week 6 | | f |
| Week 7 to 8 | | g |
| Mid-treatment measurement | | 2 |
| Week 9 to 14 | | h |
| Week 15 | | i |
| Post-treatment assessment | | 2 |
| 3 months follow-up | | 2 |
| a | Assessment of the patient’s problems and diagnosis | |
| 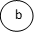 | Presentation of the treatment and discussion of the patients’ suitability and motivation | |
| c | Presentation of treatment and participants | |
| d | Psychoeducation about depression: causes and maintaining factors | |
| e | Self-assertion, interpersonal relationships, and social network | |
| f | Resources, interests, activities and friendship | |
| g | The cognitive model of depression and anxiety: automatic negative thoughts, cognitive distortions | |
| h | Thoughts and behavior, downward arrow technique, negative assumptions, analysis of benefit and countering, helpful questions to evaluate negative thoughts | |
| i | Relapse prevention and evaluation of treatment | |
| 1 | Patient’s workbook | |
| 2 | Beck Depression Inventory 2^nd^ ed. and Beck Anxiety Inventory | |
